# Embarking on an academic career with online teaching: changes in self-efficacy and its sources

**DOI:** 10.3389/fpsyg.2026.1797868

**Published:** 2026-09-08

**Authors:** Jingjing Dong, Yuanyuan Ren

**Affiliations:** 1 School of Education, Shaanxi Normal University, Xi’an, Shaanxi, China; 2 School of Educational Science, Yancheng Teachers University, Yancheng, Jiangsu, China; 3 School of Human Sciences, Tarim University, Aral, Xinjiang, China

**Keywords:** early-career academics, novice teacher, online teaching, teacher education, teacher self-efficacy

## Abstract

**Introduction:**

Commencing teaching is overwhelming, and more so with transitions in teaching formats. Teacher self-efficacy (TSE), situated at the center of teacher motivation, can affect both teacher and student performance; however, it resists change once established in the initial stage. Therefore, it is essential to examine its flexibility and the sources that influence it in this period. Few studies have been conducted on the TSE of academics who embarked on their career with online teaching related to COVID-19.

**Methods:**

This mixed-method study examined changes in online teaching self-efficacy (OTSE) of 69 first-year academics measured at the beginning and end of online teaching during the lockdown retrospectively, out of whom four were interviewed for OTSE sources.

**Results:**

TSE for online teaching (*β* = 0.69, *p* < 0.001) and technology application (*β* = 0.74, *p* < 0.001) increased significantly. Mastery experience, including prior teaching-related experience, less demanding characteristics of online teaching, subject and pedagogical knowledge, and perceived students’ performance, was the primary source. Verbal feedback from colleagues and students, and initial stress emotions were the other two main sources. Vicarious experience was rarely reported.

**Discussion:**

Higher education institutions could explore strategies, including the provision of hands-on experience and gradually increasing early-stage workload to prepare their staff for innovative teaching contexts like online and blended teaching. Limitations and suggestions on future research are discussed.

## 1 Introduction

The first year of teaching is one of the most stressful stages of the teaching career, witnessed by severe dropouts and mental health conditions of early-career teachers (George et al., 2018) due to various challenges. These challenges include adjusting to independent teaching responsibilities, administration duties, and balancing professional and personal pressures, potentially resulting in adverse effects, such as failing to develop confidence in teaching effectively (González-Calvo et al., 2020). Such an experience is particularly applicable to early-career academics who have been trained to be scientists rather than teachers (Brown et al., 2025), and they were recruited and appointed mainly based on their research capability (Douwes-van Ark et al., 2025). First-year teachers’ initial judgments on individual teaching capability, termed teaching self-efficacy (TSE), play a decisive role in forming a capable self-image in their upcoming career. For instance, novice teachers’ robust TSE could protect them from emotional exhaustion and depression, which, in turn, could strengthen their commitment to the teaching profession (Chesnut and Burley, 2015). Although TSE has been extensively studied among both pre- and in-service teachers at school levels, there have been few studies on TSE among novice academics (Douwes-van Ark et al., 2025), despite their potential to experience significant challenges and a general lack of pre-service training on teaching (Aldrup et al., 2020).

The present study investigated a cohort of first-year academics who embarked on their careers with online teaching due to social restrictions caused by COVID-19. Limited studies have examined how novice academics’ perceptions of their online teaching capability changed, although they would have encountered obstacles during this period (Moorhouse, 2021). It is particularly essential considering the context specificity of self-efficacy (Bandura, 1997), indicating that teachers evaluated their TSE differently across different teaching levels and teaching (Douwes-van Ark et al., 2025; Perera et al., 2019). For instance, although classroom management has been regarded as one of the most concerning domains of school teaching, academics rated student engagement and course development as their top priorities (Brown et al., 2025). Also, theoretically, self-efficacy experiences the most flexibility during the initial developmental stages or periods of newly experimenting innovative teaching methods (Bandura, 1997) so it becomes necessary to explore changing trajectories of academics’ TSE, particularly for the unprecedented online teaching. To the researchers’ best knowledge, rare study has investigated the changes in the first-year academics’ online teaching self-efficacy (OTSE) and the sources that contributed to such. In addition, it is essential to investigate their OTSE, given international trends toward integrating online and physical teaching in higher education, which have raised innovative expectations for their teaching (Han and Gao, 2023; Hill, 2025). Therefore, this study used a mixed-methods approach to investigate changes in OTSE among a cohort of first-year academics who began their careers in online teaching due to COVID-19 restrictions.

## 2 Literature review

### 2.1 Early career academics’ teaching-related experience and self-efficacy

Although an academic career involves more than just research (Boyer, 1990), academics tend to apply their main efforts to achieving publications rather than teaching and other domains like social services (Brown et al., 2025; Udegbe, 2016). This intention is evident among many early-career academics who are concerned about establishing themselves in their research field or securing a permanent position through conducting research (Tong, 2020). For instance, most early-career academics have reported research as the key to career success, with approximately three-quarters stating that teaching was not their preference (Matthews et al., 2014), even though they felt underprepared to complete the scheduled content, conduct in-class activities, and rate students’ performance (Udegbe, 2016). Meanwhile, it is not uncommon to witness the neglect of training in teaching in research degrees worldwide (Greer et al., 2016). Only 16% of 12,000 higher research degree students in Australia reported having participated in training on teaching (Edwards et al., 2011). The percentages in the UK and US were approximately 30 and 50%, respectively (Connolly et al., 2018; Greer et al., 2016). In China, there is no systematic teaching training in the higher education system (Tong, 2020).

Early-career academics often express uncertainty about teaching due to insufficient preparation (González-Calvo et al., 2020), which could adversely affect their professional development. Such an individual perception of their capability to accomplish specific tasks, termed self-efficacy, could influence their goal-setting, persistence in the face of adversities, and mental health (Bandura, 1997). This construct has been exclusively investigated among school teachers considering its impacts on teachers’ disposition, behaviors, and student achievement (Zee and Koomen, 2016). However, TSE of academics has received limited attention (Hill, 2025; Matos et al., 2022), which does not appear to have changed since the original call was made for more studies on TSE among academics (e.g., Landino and Owen, 1988). Certain research indicated academics appearing to be confident in different aspects of teaching, such as tutorials, seminars, and lectures (Sharp et al., 2013); however, early-career academics were not the focus of the study. Recent evidence on the impacts of academics’ TSE has been accumulated. Their TSE for instructional strategies significantly predicts the capability of academics to handle challenges in differentiating tasks and commenting on students with varying needs (König et al., 2020), their attitudes, and job satisfaction (Matos et al., 2022). It also mediated the impact of faculty resource and autonomy support on academic emotional exhaustion and working engagement (Han and Gao, 2023). Furthermore, self-efficacy is flexible in its early stages of development and resists change once established (Bandura, 1997), and TSE has been reported to tend to plateau after its early-year developments (Klassen and Durksen, 2014). Therefore, it is necessary to investigate the changes in academics’ TSE in early career which could contribute to high teaching quality and teacher well-being together with positive student academic performance (van Dijk et al., 2024a, b).

### 2.2 Changes in early career academics’ TSE and its sources

Among the limited studies investigating changes in TSE among academics, doctoral students reported uncertainties about teaching effectively; however, their TSE improved through participating in a program that integrated community activities with academics who had teaching experience (Greer et al., 2016). Similar concerns were reported by academics about teaching in their initial years (González-Calvo et al., 2020). For instance, first-year academics had low TSE for preparing syllabi, aligning their instruction with syllabi, and managing students (König et al., 2020; Udegbe, 2016). However, Edwards et al. (2011) found that 50% of their participants (*N* = 12,000) reported feeling highly prepared, and 42% were moderately satisfied with their teaching capability. Also, academics reported being self-efficacious across different domains; however, their TSE varied across domains of varying complexity. For instance, being lowest in TSE for differentiation and student assessment (Douwes-van Ark et al., 2025).

High TSE, also noteworthy, is not always preferable as less-seasoned teachers might make unrealistic positive judgments due to the lack of matured understanding of the teaching complexity. In contrast, low TSE might stimulate individuals’ reflection, which, in turn, brings development (Noben et al., 2021). Although much has been reported on TSE levels, less is known about whether/how it changes across the initial stages of their academic career (van Dijk et al., 2024a, b). The latter becomes essential as it is challenging to see changes in TSE after its early development stages (Klassen and Durksen, 2014).

Research on TSE sources, therefore, has been regarded as essential, but limited, to delineate TSE formation and change, which, in turn, facilitates teacher professional development (Yin et al., 2020). Self-efficacy change becomes possible because of the interplay among four sources: mastery experience, vicarious experience, social persuasion, and physiological and affective states (Bandura, 1997). According to Bandura (1997), mastery experience informs individuals’ beliefs in their capacity based on their previous failures and successes and is the most resilient source of self-efficacy. Individuals’ judgments on their capability could also be made by observing whether similar others could complete the tasks, labeled vicarious experience. Also, the information provided by trustworthy others could change individuals’ conception of their capability, and such judgment might differ across emotions, corresponding to social persuasion and physiological and affective states, respectively. However, research on TSE sources has encountered certain obstacles, including a lack of validated measurements that are theoretically aligned, being ambiguous about the underlying mechanism of how different sources inform the formation of TSE, and the existence of additional sources like pedagogical knowledge (see Morris et al., 2017). Therefore, mixed-method designs could be utilized to combine TSE changes measuring with qualitative information on the sources (Matos et al., 2022).

Lacking teaching preparation was reported as having made early-career academics uncertain about teaching larger classes (González-Calvo et al., 2020). In contrast, prior teaching-related experience in either informal teaching or pre-service training contexts is beneficial for early-career academics’ TSE (Hemmings, 2015) by familiarizing them with the strategies needed for university teaching (Greer et al., 2016). For instance, working in the laboratory assisted early-career academics to feel better about laboratory teaching. Other than previous experience, characteristics of mentoring, such as time adequacy, frequency of meeting, and time spent with mentees, could explain more than half of early-career academics’ reports on the improvements in their teaching (Kwok et al., 2021). For instance, academics who were accessible to peer assistance and provided with more autonomy to make decisions tended to report a higher TSE (Han and Gao, 2023). However, research on the effectiveness of such induction programs has predominantly focused on school teachers who teach in physical classrooms. In contrast, inductions provided to support early-career academics focus more on research (Connolly et al., 2018), which inhibits the opportunities for early-career academics to improve their teaching capability.

Both vicarious experience and social persuasion were rarely mentioned as influential by academics because having limited opportunities to either observe or receive advice from their colleagues (Noben et al., 2021). Modeling colleagues’ teaching or that embedded in teaching training was declared mainly positively effective on academics’ TSE, whereas both positive and negative effects of persuasive information were reported (Myyry et al., 2022). In addition, physiological and affective states were less reported (Noben et al., 2021). Yin et al. (2020) found a low but significant correlation between stress, ranging from that related to the financial shortage and adoption of new technologies to teaching-research conflicts and TSE. Also, the association varied across different domains of academic TSE, with TSE of course design and student management being positive, whereas that with TSE of student management being negative (Han and Gao, 2023). However, it is unclear whether physiological and affective states could contribute to the TSE of first-year academics independently because those who are less experienced tend to report a low TSE and stress simultaneously (González-Calvo et al., 2020). In contrast, TSE has been reported to protect academics from work stress (Pei et al., 2024).

### 2.3 Early career academics’ online teaching self-efficacy related to COVID-19

Very few studies have been conducted on early-career academics’ reactions to online teaching, especially those who have just embarked on their career with this teaching format (Han and Gao, 2023; van Dijk et al., 2024a,b). As to studies based on school teachers, the majority of first-year school teachers, who started their career with online teaching amid Covid-19, reported they were not well prepared to teach remotely; however, they were confident with their subject knowledge (Moorhouse, 2021). Interestingly, novice school teachers in Scotland reported a higher level of TSE for online teaching than for physical teaching (Carver and Shanks, 2021). However, no sufficient arguments for the reasons of such a finding, as only quantitative data on TSE levels were collected (Carver and Shanks, 2021). Regarding early-career academics, they tend to be least confident in their ability to engage students compared with other aspects of teaching, including online instruction, technology integration, classroom management (Horvitz et al., 2015), and organizing assessments of students in online contexts (Hemmings, 2015). However, they might commence their careers with advantages in coping with digital technologies compared with their veteran colleagues (Prensky, 2001).

Research has also been conducted on academics’ experiences related to transferring higher education courses to online teaching during Covid-19. For example, more than 80% of academics felt incapable of adjusting the content delivery format and assessing their students’ attention, and most respondents reported limitations associated with using online platforms to effectively organize small learning groups (Casacchia et al., 2021). However, increases in academics’ OTSE in studies (e.g., Myyry et al., 2022) tracked them through two-month online teaching during Covid-19. Conversely, Watermeyer et al. (2021) reported that over 60% of academics were efficacious about online teaching and conducting students’ performance assessments. Both faculty assistance and fewer challenges in online teaching, such as the reduced time spent on lesson preparation, were reported to have influenced their TSE (Watermeyer et al., 2021). In addition, purposefully designed professional development for online teaching has been reported to have increased academics’ TSE for online teaching. That featured with addressing peer empathy and meaningful student-teacher communication increased TSE for instructional strategies, technology usag and online learning environment creation (Hill, 2025).

Lacking experience, either in the physical classroom or online teaching, and having insufficient knowledge of digital technology were among the most frequently reported influential factors of academics’ OTSE (Horvitz et al., 2015). Furthermore, the number of semesters taught online was positively and significantly associated with OTSE for classroom management, indicating that longer experience dealing with students assists the increase in academics’ self-efficacy (Horvitz et al., 2015). However, unsuccessful online teaching experiences of early-career academics do not necessarily result in low TSE because teachers can work through students’ disengagement with suggestions from colleagues and, thereby, improve their OTSE (Xu, 2021). Furthermore, student learning performance has been reported to have the most significant impact on online teaching self-efficacy (Horvitz et al., 2015). However, misunderstanding online teaching content and students’ disinterest discourage early-career academics from maintaining a high level of OTSE (Hemmings, 2015). Also, early-career academics value positive verbal persuasions from their students and colleagues, and this positive feedback positively impacts their OTSE (Hemmings, 2015). However, the effects of positive comments might depend on teachers’ intentions to explore and openness to potential suggestions (Xu, 2021). In addition, the OTSE of first-year school teachers improves after one semester of online teaching because of being familiar with school administration and engaged with the school community (Moorhouse, 2021). It could be because such positive emotional states increased the likelihood for first-year academics to feel positive about their capabilities (Klassen and Durksen, 2014), considering the pleasant emotion and energetic feelings associated with working engagement significantly predicted academic TSE for online teaching (Huang et al., 2022).

### 2.4 Research gap and the present study

There is a paucity of studies on academics’ self-efficacy for teaching, and the sources that influence it, and existing studies have relied on cross-sectional designs (Brown et al., 2025; Noben et al., 2021; Myyry et al., 2022). When reviewing this topic, we could not find any research on OTSE of first-year academics who started their career with online teaching during the interruption of Covid-19. Therefore, this current study aimed to apply a retrospective pre-and post-design to investigate changes in a cohort of first-year academics’ OTSE, who started their career with online teaching before returning to physical classroom teaching approximately six weeks later. In addition, one-on-one interviews were conducted to triangulate the quantitative data and explore the sources influencing OTSE changes.

## 3 Method

### 3.1 Participants

Participants were first-year academics (*n* = 69) at a local teaching-oriented university in Jiangsu province, China, who had either a master’s or a doctoral degree. All participants commenced the teaching semester online at the end of August 2021, and online teaching continued for almost six weeks due to the unexpected identification of positive Covid-19 cases in the province. Classes resumed physical teaching in the middle of October 2021. Participants received induction training over the first two weekends of October (four weeks after commencing teaching), which mainly included content on the university’s policies for promotion, research grant application processes, regulations on teaching assessments, and two exemplary courses on intelligent classroom teaching given by two experienced teachers. The training was given by leaders and directors from the different departments of the university.

### 3.2 Questionnaire

#### 3.2.1 Scale for online teaching self-efficacy

The scale used to measure OTSE consisted of two sections. The first section focused on teaching in an online context, focusing on promoting students’ deep learning (see Filius et al., 2018; Gosselin, 2009). Nine items were created to cover three aspects of OTSE, namely enhancing the engagement of students (e.g., reasonably distributing learning tasks to my students and instructing them to study cooperatively), addressing critical thinking (e.g., integrating inspiring questions into my online teaching to guide students to think critically), and encouraging students’ learning autonomy (e.g., address the essence of managing study time independently). Participants were instructed to answer using a five-point response scale ranging from always (5), often (4), sometimes (3), occasionally (2), to never (1). In a pilot analysis, the one-factorial structure received a satisfactory model fit, namely χ2/df = 1.96. CFI = 0.975, TLI = 0.963, RMSEA = 0.076. SMRM = 0.0406, and in the present study, had good internal consistency (*α* = 0.979 at Time 1; α = 0.979 at Time 1). The second section contained three items measuring self-efficacy for online technology applications taken from the validated Scale for Online Teaching Self-Efficacy (Ma et al., 2021), and had good internal consistency in the current study (α = 0.961 at Time 1; α = 0.962 at Time 1). The decision to only include items on technology application was made considering that the instructional dimension of the scale was rooted in school teaching.

#### 3.2.2 Demographic variables

Demographic information collected included gender, prior teaching experience in higher education (if so, whether longer than three years), and discipline. Other questions related to the first-year experiences of participants were included, such as what tasks took the most of their time and energy, how satisfied they were with the induction measures taken by their department, whether the induction provided by their college was systematic and high-quality, and whether they felt confident transferring to physical teaching. An invitation for participation in a semi-structured interview was included at the end of the survey, and those who wanted to be interviewed could leave their contact details.

### 3.3 Data collection

An invitation to complete online questionnaires was administered in the social media group organized for first-year academics during the week after the commencement of physical teaching in October 2021. The questionnaires were available for the first two weeks of physical classroom teaching. The online questionnaire included information on demographic details, questions related to first-year teaching experiences, and the scale for OTSE. Participants were instructed to retrospectively report their OTSE at the start and the end of the online teaching period (i.e., two sets of responses). Notably, this retrospective method has advantages in data acquisition efficiency, its simplicity (Jordan, 1967), and maintaining participants over time (Euser et al., 2009); meanwhile, such a retrospective method has been questioned about causing inaccurate estimation of the construct at the earlier timepoint. Despite empirical evidence confirming its advantages in assisting participants with making consistent judgments on the constructs, especially considering the mature understanding of the construct at a later time (Little et al., 2020).

Each of the four participants who consented to be interviewed was contacted, and those who accepted the invitation were interviewed in the office of the first author in the week following the completion of the survey. Each interview lasted 40–60 min and was audio-recorded before being transcribed. Interviewees were asked about their understanding of being an academic, how they perceived their capability to teach online before and after the online teaching experience, what potential influential factors influenced their judgment, how they felt about transferring to physical teaching, and a list of potentially influential factors, including induction and mentoring, that impacted their OTSE.

### 3.4 Data analysis

Quantitative data were analysed in SPSS Version 23 (IBM Corp, 2021). First, descriptive analyses were conducted. Four-step multilevel modeling was then performed to investigate the changes in OTSE and influential factors. In the first model, a null model was tested to examine whether multilevel modeling was warranted by checking the intraclass correlation coefficient (ICC). Then, time was set as the only explanatory factor of the within-individual variation to check whether/how OTSE changed across time. The third model included demographic variables as explanatory factors to adjust for initial differences in TSE levels attributable to the participants’ background information. In the fourth model, only demographic variables that had elicited significant effects on changes in OTSE were retained to explore their impact. Both −2 times log-likelihood (−2LL) and Akaike’s information criterion (AIC) were used to indicate model fit, with smaller values indicating better model fit. The significance of -2LL was tested based on the chi-square distribution, and a reduction of not less than 6 in AIC refers to a significant improvement in the model fit (Heck et al., 2013). Results are reported based on the best-fit model.

Qualitative data from the interviews were analysed in NVivo 12 (Lumivero, 2020) by applying a five-step procedure (Braun et al., 2016): data familiarization, generating initial codes, identifying and reviewing themes, refining the coding and theme frameworks, and reporting the results. At first, all interview recordings were transcribed by a certified company. Then, the first author read the transcripts repeatedly to familiarise himself with the data and form an overall understanding of it. The author focused on developing an initial interpretation of each interview’s transcript during this process. Second, initial codes were generated via a reflexive iteration. This process allowed the researcher to interpret the data with the research questions in mind and was only suspended when no more codes could be identified. Third, themes such as four sources of TSE, namely mastery experience, vicarious experience, social persuasion, and physiological and affective states, were created, followed by coding different information into each theme. These themes were refined by removing any code displaced prior to being reported as to the research questions.

## 4 Results

### 4.1 Quantitative data

#### 4.1.1 Descriptive findings

In total, 69 participants were included in the final analysis (Table 1). Slightly more than half, 36 (52.2%), were male academics, 38 (55.1%) were from science and social science disciplines. Thirty (43.5%) were married, and 38 (55.1%) had a master’s degree. Forty-three (62.3%) participants reported they spent most of their time on teaching. Half of the participants (35, 50.7%) thought teaching and research were both important but energy-consuming.

**Table 1:** Descriptive characteristics of participants.

Variable	*n*	%
Gender		
Female	33	47.8
Male	36	52.2
Research discipline		
Science	19	27.5
Social science	19	27.5
Engineering	14	20.3
Humanity	10	14.5
Agriculture	2	2.9
N/A	5	7
Marital status		
Married	30	43.5
Single	39	56.5
Degree		
Doctorate	31	44.9
Masters	64	55.1
Time spent most		
Teaching	43	62.3
Research	10	14.5
Family	11	15.9
Others	5	7.2
Pressure balancing		
Neither teaching nor research^a^	35	50.7
Teaching rather than research^b^	13	18.6
Well balanced^c^	8	11.4
Research rather than teaching^d^	5	7.2

^a^Teaching and research matters equivelantly but had limited energy; ^b^ teaching is too pressuring to focus on research; ^c^ teaching and research were being balanced well; ^d^ research is too pressuring to focus on teach.

Means, standard deviations, skewness, kurtosis, and correlation of the OTSE subdomains and other variables are listed in Table 2. Values of both skewness and kurtosis lie within a normal distribution. Two OTSE domains correlate significantly across two timepoints, ranging from 0.579 to 0.702. Both confidence in physical teaching and induction satisfaction negatively correlate with both domains of OTSE across two timepoints, ranging from −0.502 to −0.226. These two variables positively correlated (r = 0.27, *p* < 0.005).

**Table 2:** Description of mean, skewness, kurtosis and correlation coefficient between online teaching self-efficacy, and variables.

Variables	Online teaching 1	Online teaching 2	Technology 1	Technology 2	Confidence in physical teaching	Satisfaction with induction
1 Online teaching 1^a^	1					
2 Online teaching 2^b^	0.702**					
3 Technology 1^c^	0.949**	0.667**				
4 Technology 2^d^	0.579**	0.903**	0.596**			
5 Confidence in physical teaching	−0.495**	−0.502**	.-0.481**	−0.501**		
6 Satisfaction with inductiont^e^	−0.226**	−0.404**	−0.23**	−0.442**	0.265*	1
Skewness	0.68 (0.29)	−0.18 (0.29)	0.47 (0.29)	−0.24 (0.29)	0.11 (0.29)	0.74 (0.29)
Kurtosis	−0.07 (0.57)	−0.21 (0.57)	−0.40 (0.57)	−0.42 (0.57)	−0.07 (0.57)	−0.53 (0.57)
Means	1.98	2.73	2.05	2.74	2.29	1.58
SD	0.819	0.772	0.868	0.836	0.859	0.673

^a^Online teaching 1 indicates TSE for online teaching at Time 1; ^b^Online teaching 2 indicates TSE for online teaching at Time 2; ^c^Technology 1 indicates TSE for technology application at Time 1; ^d^Technology 2 indicates TSE for technology application at Time 2; ^e^Satisfaction with the induction measures taken by their department. *p < 0.05, **p < 0.01.

#### 4.1.2 Changes in online TSE and influential factors

The unconditional models of both OTSE domains produced ICCs of 0.624 and 0.601, indicating 62.4 and 60.1% variance is related to time, respectively. The assumption that OTSE is independent of time measurement is violated. In other words, multilevel modeling was warranted. Model fits of both domains of TSE improved significantly in model 2 with *Δ* AIC > 6 and Δ -2LL > 3.84 with one degree of freedom (Table 3). Significantly individual differences were found in both TSE for technology and online instruction at Time 1 (*p* < 0.001). Regarding changes over time, OTSE for online teaching increased significantly between Time 1 (M = 1.98, SD = 0.819) and Time 2 (M = 2.73, SD = 0.772) (*β* = 0.74, t (68) = 10.0, *p* < 0.001, 95% CI [0.594, 0.890]). OTSE for technology application also increased significantly between Time 1 (M = 2.05, SD = 0.87) and Time 2 (M = 2.74, SD = 0.84) (β = 0.69, t (68) = 7.432, *p* < 0.001, 95% CI [0.502, 0.870]). A relatively large proportion of variance in both OTSE domains was explained by the time variable, namely 31.8 and 43.1%, respectively (Table 4).

**Table 3:** Characteristics of interviewees.

Interviewees	Gender	Degree	Informal experience	Research field	Teach 1^a^	Teach 2^b^	Tech 1^c^	Tech 2^d^
Helen	Female	Doctorate	Yes	Early childhood development	2	1.67	2	1.67
Julia	Female	Master’s	Yes	Primary education	1.89	2.44	2	3
Yaretzi	Female	Doctorate	No	Intelligence and creativity	1.67	2.56	2.3	2.3
Zara	Female	Master’s	Yes	Clinical psychology	1.56	2	2	2

^abcd^Teach 1 and Teach 2 indicate TSE for online teaching at Time 1 and Time 2, namely at the beginning of and six weeks after online teaching period; Tech 1 and Tech 2 indicate TSE for online technology application at Time 1 and Time 2, namely at the beginning of and six weeks after online teaching period.

**Table 4:** Model parameters and goodness of fit for multilevel modeling of OTSE.

Parameters	Online teaching^a^		Technology application^b^	
	Model 1	Model 2	Model 1	Model 2
Fixed effects(is)				
Intercept	2.74**	3.37**	2.73**	3.31**
Time 1	−0.69**	−0.69**	−0.74**	−0.74**
Time 2^c^				
Male		0.32		0.306
Female^d^				
Married		−0.046		−0.085
Not Married^e^				
Master		−0.152		−0.172
Doctorate^f^				
Satisfaction^g^		−0.442**		−0.387*
Random effects				
Residual	0.294**	0.294**	0.190**	0.190**
Intercept	0.432**	0.34**	0.444**	0.37**
Model summary				
AIC	325.173	316.646	290.514	283.942
−2LL	321.173	312.646	286.514	279.942
Number of parameters	4	11	4	11

^a^Online teaching indicates TSE for online teaching; ^b^Technology application indicates TSE for technology application; ^cdef^The parameter is redundant; ^g^Satisfaction indicates satisfaction about induction; ^g^Satisfaction indicates satisfaction about induction. *p < 0.05, **p < 0.01.

Regarding influential factors, only the induction quality significantly predicted changes in both subdomains, namely TSE for online instruction (β = −0.548, *p* < 0.001) and that for technology application (β = −0.408, *p* < 0.001).

### 4.2 Qualitative data

#### 4.2.1 Characteristics of interviewees

Four survey participants accepted the invitation and completed a one-on-one interview (see Table 3). They were all females and in their late thirties or early forties.

#### 4.2.2 Sources of online teaching self-efficacy

##### 4.2.2.1 Mastery experience

Prior teaching relevant experience: All interviewees reported having some teaching-related experience, which, in turn, benefited their certainty about teaching online to varying extents. Helen reported a moderate initial level of TSE, which was mainly attributed to her experience in teaching the same subject part-time at the university for one semester. Also, Yaretzi did not feel challenged starting her career with online teaching because of her online presentation experience during her doctoral studies. In contrast, although Zara had taught the same subject for one year in a vocational college, she felt uncertain about teaching effectively in the current university because the students in the college were less academically interested. It seems that the informing of existing experience influenced their judgment of self-efficacy; however, its relevance, including online-associated and student comparability, moderated the extent of such effects.

Online teaching being less demanding: Of many surprises, all interviewees considered online teaching to be less challenging compared with physical teaching. Two main reasons were given for this. First, students were predominantly quiet during online teaching so that interviewees, as to Zara, were “mainly talking to the screen and did not have to respond to the students.” However, interviewees did not have to react to this silent online context. In contrast, they were forced to make constant adjustments in the physical classroom, even though their students were not reactive, because they could sense students’ involvement and understanding just by observing their facial expressions. Second, interviewees reported being able to “delay in response” in an online context, whereas they had to continuously speak to engage students in physical classes. Therefore, the challengeable nature of online teaching itself provides the criteria based on which the interviewees made their self-efficacy evaluation, and it differs from TSE for traditional teaching in higher education.

Subject and pedagogical knowledge: TSE varied with interviewees’ perceived knowledge of different subjects. On the one hand, Zara, who earned a master’s degree in psychology, felt very confident in teaching introductory psychology. However, she felt inadequate teaching management psychology because it is “quite different from my knowledge background.” On the other hand, both Helen and Yazetzi held doctorates in psychology and felt comfortable integrating their research into their teaching by updating the textbook and providing additional knowledge. However, all interviewees realized that the differences between content and pedagogies are in knowledge. For instance, Yazetzi said that “it is completely different between knowing the knowledge and how to teach it.”

Students’ performance: How to engage students was reported by all interviewees as challenging during online and physical classroom teaching, which was primarily applicable to online teaching. As Helen explained, “students were quiet and did not answer even when I raised questions.” However, making progress in motivating students enhanced the teachers’ OTSE. For instance, students from one of Julia’s classes enrolled in a program that secured a permanent position for students once they graduated, were less motivated to participate in the class. Still, Julia felt they were becoming more active and showing no difference from her other classes with her efforts to engage. In addition, interviewees’ TSE might be less robust as they mainly considered their capability to engage students at this early stage and failed to report on other forms of student performance.

##### 4.2.2.2 Social persuasion and vicarious experience

Feedback from colleagues and students: All interviewees reported receiving informal advice, either from department leaders or other experienced teachers, as influential on their certainty about teaching. However, the effects of such appeared to be limited. Zara received comments on her teaching; however, these were regarded as general and not specific. Also, she pointed out that leaders and colleagues tended to ‘praise’ you rather than point out where you could improve, considering their feedback to be more friendly. Therefore, it appears that the positive, comforting words of their colleagues were not considered informative; in other words, persuasion shall be designed to be constructive and specific to be informative.

Another type of ‘feedback’ related to students was considered more frequently and valued by interviewees and was particularly obvious when participants reflected on the transition from online to physical classroom teaching. For instance, all of them noticed students’ involvement in the class through students’ facial reactions, which, in turn, impacted their judgment of their capability to engage students.

Observing experienced colleagues: All participants also commented on their willingness to follow and copy an experienced teacher who shares the same subject. They were desperate to learn, as to Helen, “how the experienced teachers teach and use it in my teaching.” However, none of them could observe the other’s teaching, and only Helen was shared the recorded courses of experienced colleagues.

##### 4.2.2.3 Physiological and affective states

Initial stressful emotions: All interviewees felt overwhelmed, and none reported TSE higher than 2 out of 4 in the survey. Stressors included a lack of time to prepare the lesson, coping with unexpected administration, research-related workload, and socializing with colleagues, which threatened their certainty about teaching effectively. First, all did not know what subjects they were to teach for roughly the first couple of weeks before the new semester started. As Zara reflected, “Everything was so nervous and uncertain. I only knew the teaching subject about three or four days before the semester started.” Second, much time is needed to prepare lessons throughout the online teaching period. Yaretzi, who had no prior teaching experience, felt it was tiring preparing lessons from Monday to Friday, even though she only taught two classes a week. Third, tasks other than teaching, like research, administration, and family issues, took much, if not the majority, of their time and energy, and this impacted their perception of teaching capability. Helen, the only interviewee whose two TSE domains decreased (Mteach1 = 2, Mteach2 = 1.67; Mtech1 = 2, Mtech2 = 1.67), was overwhelmed by extra research projects assigned by the directors and rarely had time to prepare her lessons. The theme indicates that non-teaching-related workload comes into effect through worsening first-year academics’ emotional states. Also, this report could indicate the interaction of different sources like mastery experience and emotional states.

## 5 Discussion

This mixed-method study applied a retrospective pre-and post-survey, followed by in-depth interviews, and investigated OTSE change of first-year academics at a Chinese university, and its sources. At first, both quantitative and qualitative results indicated an initial lower OTSE at Time 1, namely the beginning of the semester, compared to roughly six weeks later at Time 2, which aligned with González-Calvo et al. (2020), who found that early-career academics experienced many doubts over their teaching capability. Regarding the changes in OTSE, quantitative results indicated that both domains of OTSE, namely self-efficacy for online instruction and technology application, increased significantly after the online teaching period, aligned with the findings of Moorhouse (2021), and confirmed the predominant role of mastery experience proposed by Bandura (1997). Such findings were also triangulated by qualitative data, which indicated that first-year academics did not regard online teaching to be as challenging as physical classroom teaching, contradicting the reports on the former being more confronting (Casacchia et al., 2021). This finding, however, confirmed the findings of Hemmings (2015) that certain features of online teaching, like not having to respond to students spontaneously, reduced the pressure of early-career academics. Compared with reports of online teaching being challenging, due to factors including separating from students (e.g., Casacchia et al., 2021), interviewees in the present study reported having benefited from such features, which reduced the complexity of teaching.

Regarding qualitative reports of first-year academics being uncertain about transferring into physical classroom teaching, this study failed to confirm the findings of Sharp et al. (2013) that academics tend to have a high TSE for different domains of teaching. This is because all interviewees reported having experienced transitioning difficulties when facing a large cohort of students, engaging students via talking continuously, and delivering the knowledge in an approachable way, as indicated in other studies (e.g., Horvitz et al., 2015; König et al., 2020). However, the results of the present study align with prior studies on first-year school teachers (e.g., Carver and Shanks, 2021; Moorhouse, 2021), with first-year academics feeling less self-efficacious about transferring into physical classroom teaching.

Despite the OTSE increases in quantitative data, the interviewees expressed their concerns about the validity of their TSE, mainly considering their lack of cognition about the effects of their teaching. This is consistent with Bandura's (1997) theory that the outcome of experiences plays a decisive role in individuals’ perceptions of their capability. This finding might have also resonated with the reports on prior teaching experience by interviewees, who reported benefits from this prior experience, especially those being authentic and generalizable into their teaching. This finding contradicts prior studies (e.g., Udegbe, 2016) that early-career academics failed to benefit from their prior experience, resulting in low TSE. This contradiction might be explained by the characteristics of experience reported in the current study, such as being extremely relevant to the teaching subject and going through difficulties, aligned with Greer et al. (2016). Therefore, compared with the findings of Hemmings (2015), the current interviewees benefited much from the prior experience because of its relevance to their teaching, which also confirmed the vital generalizability of mastery experience (Bandura, 1997).

Among the influential factors of OTSE changing, the level of satisfaction with the induction program was reported to be important in both the quantitative and qualitative data. This is consistent with the findings of Kwok et al. (2021), that well-designed induction programs can play a significant role in developing the teaching capability of early-career academics. However, there appeared to be a contradiction between the quantitative and qualitative findings in the quality of induction. The high level of quality reported in the quantitative data might be because participants rated this in terms of different spheres, including research and teaching. This might resonate with the qualitative findings that the induction was mainly focused on research and administration-related aspects, aligned with the findings of Connolly et al. (2018), making participants doubt the value of such for assisting them to teach effectively. One other reason for the positive reports on induction in the current quantitative data might be, as indicated in the qualitative data, that participants have benefited from the informal mentorship, which confirmed the importance of faculty support in increasing academics’ OTSE (Watermeyer et al., 2021). However, interviewees were not satisfied with such informal mentoring. Instead, all of them were keen on having a formal relationship with a mentor who teaches the same subject, for modeling and receiving feedback. This is aligned with the importance of vicarious experience and verbal persuasion (Bandura, 1997).

There were fewer reports on these two sources of self-efficacy in the present study. However, this does not necessarily mean these two sources were not important. Instead, this might indicate these first-year academics were not provided sufficient opportunities for such. Interviewees needed, but were unable to search for, mentors from the same teaching subject to model their pedagogies. However, the motivation to model mentor teachers appears to be different from what Bandura (1997) described as vicarious experience. This is because interviewees were mainly focusing on borrowing pedagogical skills from experienced teachers, rather than comparing themselves with those teachers. Second, despite participants reporting being able to receive feedback from colleagues, rare direct positive effects of such were valued in the current study, which fails to confirm Hemmings (2015) findings. This might be because not being assigned a formal mentor might have inhibited the current participants from receiving regular constructive comments. This is consistent with Bandura's (1997) theory, that the authenticity of persuasive words plays a vital role in deciding their effects on TSE. Other than pedagogical knowledge, interviewees felt teaching a subject related to their research background helped them deliver the knowledge more authentically, which could be one reason why they reported challenges in organizing the structure of courses, as reported by König et al. (2020).

Both quantitative and qualitative data indicate that the participants felt pressured in their teaching role, which discouraged them from feeling positive about their teaching capability. This confirms the findings of prior studies (e.g., Aldrup et al., 2020; George et al., 2018) that first-year academics face many competing pressures. One of the main aspects is the necessity of balancing research and teaching, aligned with prior reports of early-career academics’ preferences for achieving publications, instead of solely focusing on teaching (González-Calvo et al., 2020). This finding is also consistent with the prior finding of early-career academics failing to concentrate on teaching, due to considerations about accomplishing other tasks, including research (Matthews et al., 2014). However, the current finding could not examine the potential reciprocal association between these two constructs as initial explored by Pei et al. (2024).

## 6 Conclusion

This mixed-method study found that both TSE for online instruction and technology application increased significantly over time, which was triangulated with the qualitative data. However, qualitative data indicated participants’ TSE was challenged when they transferred to physical classroom teaching. Mastery experience was predominantly essential, followed by social persuasion and physiological and affective states, whereas vicarious experience was least essential.

Certain implications could be drawn from this research. First, a formal mentorship with a mentor from the same subject is essential to guide first-year academics to learn how to teach at a high level. This is especially important considering the lack of pre-service education in research degrees worldwide. Second, it is important to provide first-year academics with teaching resources such as previously recorded lectures in addition to encouraging first-year academics’ creativity. Third, starting with a lesser teaching load might be beneficial for first-year academics, in helping them to develop and retain a high level of TSE, considering the pressure to also conduct research. It is also important to reduce any unnecessary burden of administration-related issues. Fourth, a longer period of follow-up of first-year academics would be informative for understanding the development of early-career academics’ TSE. Fifth, theoretically, this study informs the contributions of both mastery experience and emotional states in the initial developmental period, and it also indicates the necessity of examining individual differences in changing trajectories rather than overall changes. Also, this indicates the importance of enactive teaching, given the increases in both OTSE domains, suggesting that hands-on experiences should be provided when higher education institutions prepare staff for innovative teaching methods.

Three main limitations exist in this study. First, the retrospective design might have caused inaccurate reports, although the time between the two timepoints is small. Second, the small number of participants in the surveys could limit the generalisability and power of the present study, despite the common challenges in recruiting academics into research. Third, the interviewees were selected using a convenience sampling method, and their TSE changes did not reflect those of the whole sample. Overall, this study contributed to the research on the TSE of academics by providing an initial exploration of a cohort of first-year academics embarking on their career with online teaching.

## Data Availability

The anonymized data will be made available by the authors upon reasonable request.
